# Post-radiation increase in VEGF enhances glioma cell motility *in vitro*

**DOI:** 10.1186/1748-717X-7-25

**Published:** 2012-02-22

**Authors:** Whoon Jong Kil, Philip J Tofilon, Kevin Camphausen

**Affiliations:** 1 Radiation Oncology Branch, National Cancer Institute, 10 Center Drive, Bldg 10, CRC, B2-3561, Bethesda, MD 20892, USA

**Keywords:** Radiation, VEGF, glioma cell, motility

## Abstract

**Background:**

Glioblastoma multiforme (GBM) is among the most lethal of all human tumors, with frequent local recurrences after radiation therapy (RT). The mechanism accounting for such a recurrence pattern is unclear. It has classically been attributed to local recurrence of treatment-resistant cells. However, accumulating evidence suggests that additional mechanisms exist that involve the migration of tumor or tumor stem cells from other brain regions to tumor bed. VEGFs are well-known mitogens and can be up-regulated after RT. Here, we examine the effect of irradiation-induced VEGF on glioma cell motility.

**Materials and methods:**

U251 and LN18 cell lines were used to generate irradiated-conditioned medium (IR-CM). At 72 h after irradiation, the supernatants were harvested. VEGF level in IR-CM was quantified by ELISA, and expression levels for VEGF mRNA were detected by RT-PCR. *In vitro *cancer cell motility was measured in chambers coated with/without Matrigel and IR-CM as a cell motility enhancer and a VEGF antibody as a neutralizer of VEGF bioactivity. Immunoblots were performed to evaluate the activity of cell motility-related kinases. Proliferation of GBM cells after treatment was measured by flow cytometry.

**Results:**

Irradiation increased the level of VEGF mRNA that was mitigated by pre-RT exposure to Actinomycin D. U251 glioma cell motility (migration and invasion) was enhanced by adding IR-CM to un-irradiated cells (174.9 ± 11.4% and 334.2 ± 46% of control, respectively). When we added VEGF antibody to IR-CM, this enhanced cell motility was negated (110.3 ± 12.0% and 105.7 ± 14.0% of control, respectively). Immunoblot analysis revealed that IR-CM increased phosphorylation of VEGF receptor-2 (VEGFR2) secondary to an increase in VEGF, with a concomitant increase of phosphorylation of the downstream targets (Src and FAK). Increased phosphorylation was mitigated by adding VEGF antibody to IR-CM. There was no difference in the mitotic index of GBM cells treated with and without IR-CM and VEGF.

**Conclusions:**

These results indicate that cell motility can be enhanced by conditioned medium from irradiated cells *in vitro *through stimulation of VEGFR2 signaling pathways and suggest that this effect involves the secretion of radiation-induced VEGF, leading to an increase in glioma cell motility.

## Background

Glioblastoma multiforme (GBM) is the most common and lethal primary malignant brain tumor in adults, well known for its diffusely infiltrative pattern several centimeters away from the primary disease site. Surgical removal followed by radiation therapy (RT) with chemotherapy represents standard treatment [1]. Due to the potential morbidity of whole-brain irradiation to 60 Gy, the planning target volume for RT consists of the tumor volume, defined by magnetic resonance (MR) imaging, with a 2 ~ 3 cm margin of surrounding tissue considered to be at risk for microscopic tumor invasion. However, more than 80% of untreated patients have microscopic disease within several centimeters of the contrast-enhancing tumor margin defined by computed tomography (CT) scan, and 47% of cases demonstrate histological evidence of tumor spread to the contralateral hemisphere [2,3]. This diffuse growth pattern of GBM may account for the unfavorable outcome of local therapies such as surgery and radiation, and contributes to the morbidities of both the disease and the treatment. Because the majority of tumor recurrences are found immediately adjacent to the site of resection or nearby surgical resection cavity [3,4], it has been hypothesized that radioresistance of residual tumor cells after surgical resection accounts for the local recurrence pattern. The results of recent studies, however, have demonstrated that it may also be due to changes in cellular microenvironments in the brain after treatment [5-8]. In addition, accumulating evidence suggests that molecules that are induced by primary tumors directly regulate motility in various types of malignant cells [9-14].

Vascular endothelial growth factor (VEGF), is a family of structurally related proteins, including VEGF-A, VEGF-B, VEGF-C, VEGF-D, and is essential for regulating the key steps of cell proliferation and migration. VEGF expression is up-regulated by various types of pathological conditions, malignant tumors and stresses, including surgery and RT [5-9,15]. VEGF secreted from primary tumors promotes cancer progression by inducing angiogenesis via VEGF receptors (VEGFRs) on endothelial cells but also signals directly through its receptors expressed on both cells of hematopoietic origin and a variety of tumor cells [9,12,13,16]. When VEGF binds to VEGFR, the biological effect is to cause ligand-induced dimerization and oligomerization, which activate the receptor's intrinsic tyrosine kinase activity, resulting in auto- and trans-phosphorylation on tyrosine residues in the cytoplasmic domain [17]. Enhanced VEGF expression and VEGFR activation induce malignant cell motility [10,13]. Indeed, VEGF can activate several tyrosine kinases, including Src kinase (Src), focal adhesion kinase (FAK), and paxillin [11,18-20]. Because these signaling kinases play a role in modulating cell motility [10,21-23], we evaluated the effects of IR in creating a conditioned medium that could increase tumor cell motility.

The data presented indicate that radiation-induced VEGF in cultured medium collected from irradiated glioma cells enhanced tumor motility through VEGF-stimulated Src and FAK phosphorylation. We also show that blocking the action of radiation-induced VEGF using neutralizing, anti-VEGF antibodies resulted in decreased tumor cell invasion and migration.

## Materials and methods

### Materials

VEGF_165 _and anti-human VEGF antibody were purchased from R & D Systems, Inc. (Minneapolis, NM). The anti-FAK antibodies used for Western blotting as well as the anti-paxillin were mouse monoclonal antibodies purchased from BD Biosciences (San Jose, CA). The phosphospecific rabbit antibodies against FAK at Y861 and Y925 were from Millipore (Temecula, CA) and Cell Signaling Technology (Danvers, MA), respectively, and VEGFR2 at Y996 and Y1059 from Cell Signaling Technology. Horseradish-peroxidase-conjugated secondary antibodies against mouse and rabbit immunoglobulins were from Santa Cruz Biotechnology (Santa Cruz, CA). Actinomycin D (Act D) was obtained from Sigma Chemical Co. (St. Louis, MO).

### Cell lines and tumor-conditioned medium

The U251 and LN18 human GBM cell lines were obtained from the Tumor Repository at the National Cancer Institute at Frederick (Frederick, MD). Cells were grown in Dulbecco's modified eagle medium (DMEM) (Invitrogen, Carlsbad, CA) supplemented with glutamate (5 mmol/L) and 5% fetal bovine serum (FBS), and maintained at 37 C, 5% CO_2_. To generate conditioned medium (CM), glioma cells were seeded at a density of 5 × 10^5 ^cells in 100 mm dishes. At 60% confluence, the cells were removed from the culture medium, washed three times with phosphate-buffered saline (PBS) and supplemented with 7 mL of serum-free medium. Glioma cells were then irradiated at a dose of 0.5 to 10 Gy at room temperature using a Pantak (Solon, OH) X-ray source at a dose rate of 2.28 Gy/min. After cultivation for 72 h, the supernatant of gliomas was harvested, filtered to remove debris and stored at −20 C.

### VEGF protein quantification

To quantify the VEGF protein that was released into the tumor-conditioned media of U251 and LN18 cells, the media were analyzed by a specific enzyme-linked immunosorbent assay (ELISA) kit from R&D Systems, Inc., according to the manufacturer's recommendation.

### RT-PCR and relative quantification of VEGF mRNA transcripts

After removing the supernatant from culture dishes, the total RNA of the cells was isolated using TRIzol, according to the protocol supplied by the manufacturer (Invitrogen, Carlsbad, CA); the concentration of RNA was determined spectrophotometrically at 260 nm. RNA was further purified using RNeasy Mini Kit according to the manufacturer's recommendations (Qiagen, Valencia, CA), with the addition of DNase digestion using RNase-free DNase (Qiagen), and stored at -20 C until use. Complementary first-strand DNA was generated from RNA, which was isolated from glioma cells irradiated with and without pretreatment for 1 h with Act D (5 μg/ml), using the TaqMan RT-PCR kits (Perkin-Elmer Applied Biosystems, Foster City, CA), according to the manufacturer's protocol. The PCR conditions involved an initial denaturation step at 94 C for 5 min, followed by 30 cycles at 94 C for 30 s, 55 C for 30 s and 72 C for 30 s. Primers and probes for VEGF and Glyceraldehyde-3-phosphate dehydrogenase (GAPDH) mRNA transcripts were purchased from Perkin-Elmer Applied Biosystems. PCR was performed using TaqMan RT-PCR kits (Perkin-Elmer Applied Biosystems), according to the manufacturer's protocol.

### In vitro *motility assay*

Invasion was measured using 24-well BioCoat Matrigel invasion chambers (Becton Dickinson Labware, Bedford, MA) with an 8 μm pore polyethylene teraphthalate (PET) membrane coated with Matrigel. The lower compartment contained 0.75 ml of conditioned medium or human VEGF_165 _(R&D Systems) as a chemoattractant, or serum-free DMEM medium as a control. In the upper compartment, 5 × 10^4 ^cells/well were placed in triplicate wells. For migration assays, 5 × 10^4 ^cells/well were placed in the top chamber of non-coated PET membranes (24-well insert, pore size 8-μm; Becton Dickinson Labware). The cells were incubated for 22 h and those that did not migrate through the pores in the membrane were removed by scraping the membrane with a cotton swab. Cells transversing the membrane were fixed and stained with Diff-Quick (Dade, Unterschleissheim, Germany). Stained cells were counted by light microscopy in nine randomly chosen high-power fields (x 200). Images were captured by a Photometrics Sensys CCD camera (Roper Scientific, Tucson, AZ) and imported into an IP Labs image analysis software package (Scanalytics, Inc., Fairfax, VA). Experiments were performed in triplicate.

### Neutralization of VEGF bioactivity

To analyze the VEGF effect of VEGF-neutralized conditioned medium on glioma cell motility, monoclonal anti-human VEGF_165 _was added to the conditioned medium and used for the *in vitro *motility assay (see above).

### Immunoblotting

Equal quantities of protein were separated by sodium dodecyle sulfate/polyacrylamide gel electrophoresis (SDS/PAGE) and transferred to polyvinyldene fluoride (PVDF) membrane (Millipore). The membranes were blocked with Tris-buffered saline/Tween (0.15) plus 5% dried nonfat milk for 1 h at room temperature and incubated with the desired primary antibody diluted 1:1000 in blocking buffer overnight at 4 C. Membranes were probed with antibodies to phospho-VEGFR2^Y996^, phospho-VEGFR2^Y1059^, phospho-Src^Y461^, phospho-FAK^Y861^, phospho-FAK^Y925^, Src and FAK. Primary antibody incubation was followed by incubation with a horseradish peroxidase-conjugated secondary antibody (goat anti-mouse or goat anti-rabbit) diluted 1:2000 in blocking buffer for 2 h at room temperature. Proteins were visualized with electrochemiluminescence detection reagents (Santa Cruz Biotechnology) and detected by autoradiography.

### Proliferation assay

Evaluation of cell cycle phase distribution was performed using flow cytometry. U251 GBM glioma cells were incubated for 22 h with conditioned medium or VEGF_165 _(25 ng/mL)-containing medium. Samples were fixed, stained with propidium iodide and analyzed using flow cytometry (Guava Technologies, Hayward, CA). Mitotic cells were distinguished from G_2 _cells, and the mitotic index was determined according to the expression of phosphorylated histone H3 (Upstate Biotechnology, Charlottesville, VA) detected in the 4 N DNA content population by the flow cytometric method of Xu et al. [24].

### Statistical analysis

*In vitro *experiments were repeated three times, and statistical analysis was performed using a student's *t*-test. Data are presented as mean ± standard deviation (SD). A p value < 0.05 was considered significant.

## Results

### VEGF protein quantification

**Figure 1:**
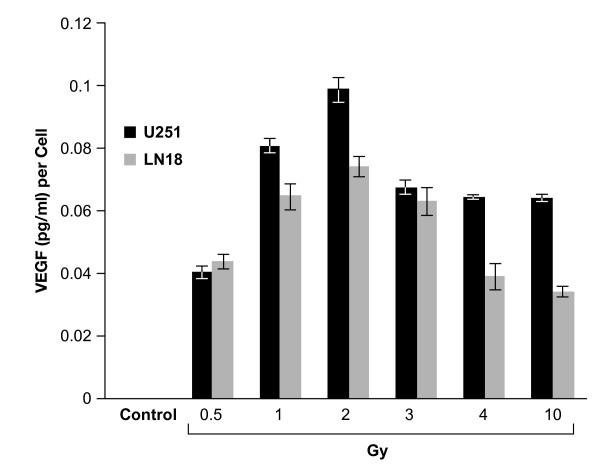
**The concentration of VEGF protein in conditioned medium**. Cells in serum-containing (5%) medium were washed with PBS and cultured in fresh serum-free medium. Culture supernatants were harvested from irradiated (0.5 ~ 10 Gy) or nonirradiated glioma cells after 72 h of incubation and VEGF protein measured by ELISA. Each value represents the mean of three independent experiments; *bars*, ± SD.

To measure the effects of IR on VEGF production in GBM tumor cells, subconfluent U251 and LN18 cells were exposed to graded doses of irradiation (IR). Three days after IR, the supernatants of each cell line were collected to generate irradiated-conditioned medium (IR-CM). VEGF levels were then measured by ELISA. The mean VEGF level in the media from unirradiated glioma cell lines was low (0.034 ± 0.003 pg/1000 cells in U251 and 0.032 ± 0.001 pg/1000 cells in LN18). In each cell line, however, a significant increase in VEGF levels after IR was measured (Figure 1). There was an IR dose-dependent increase of VEGF up to 2 Gy. At higher radiation doses, up to 10 Gy, VEGF levels in IR-CM were lower than those at 2 Gy but still remained higher than those in unirradiated supernatants. The U251 cell lines showed significantly higher VEGF secretion than LN18 cell lines treated with 2 Gy IR (98.504 ± 0.098 pg/1000 cells and 74.096 ± 0.029 pg/1000 cells, respectively; p = 0.003).

### RT-PCR and relative quantification of VEGF mRNA transcripts

**Figure 2:**
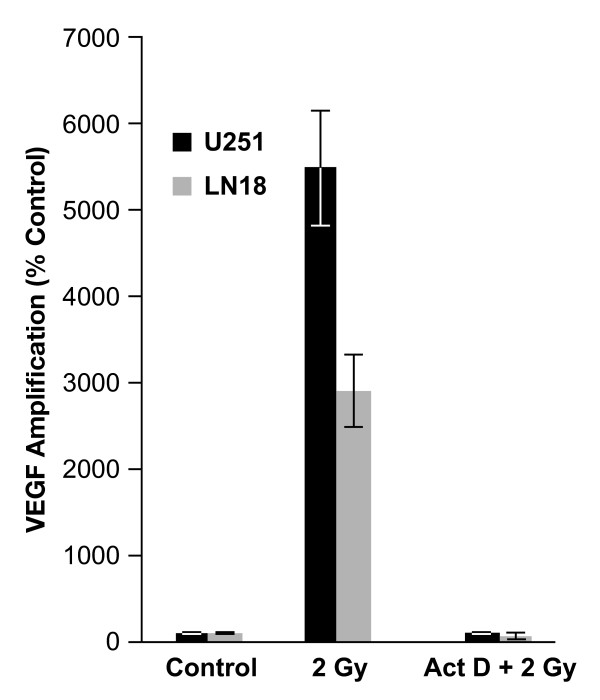
**VEGF transcription in glioma cells**. U251 and LN18 glioma cells in culture dishes with serum-free medium were pretreated with or without actinomycin. Total RNA was isolated at 72 h after irradiation (IR) and subjected to RT-PCR analysis. The data represent the mean of three independent experiments; *bars*, ± SD.

To determine whether the IR-induced VEGF increase was at the transcriptional level, VEGF transcriptions were assessed in U251 and LN18 cells by RT-PCR. VEGF transcripts were induced 55-fold in U251 and 28-fold in LN18 at 72 h after IR (Figure 2). Radiation-induced VEGF transcription was inhibited with the addition of Act D (5 μg/mL) treatment 1 h prior to IR. These findings show that IR induces VEGF expression in glioma cells due to an increase in VEGF transcription. Based on this data and the clinical relevance of the radiation dose, 2 Gy of IR was used for subsequent experiments.

### In vitro *motility assays*

**Figure 3:**
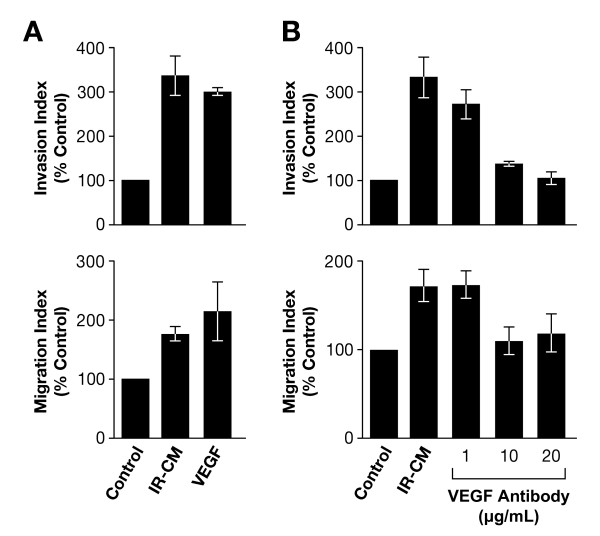
**Glioma cell motility**. U251 glioma cells were irradiated (2 Gy) and incubated in serum-free medium for 72 h, irradiated conditioned medium (IR-CM) then was collected from the supernatant of culture dishes and used for cell motility assays. Cell motility was evaluated using 24-well chambers with an 8-μm pore polyethylene teraphthalate (PET) membrane coated with matrigel for invasion assays, and non-coated PET membranes for migration assays. The lower compartment contained 0.75 ml of serum-free medium (control), IR-CM and serum-free medium with VEGF (5 ng/ml) (**A**). To neutralize VEGF activity in IR-CM, IR-CM was supplemented with monoclonal anti-human VEGF antibody (1, 10 and 20 μg/ml) (**B**). 5 × 10^4 ^cells were placed in top chamber, incubated for 22 h and glioma cells transversing the membrane were fixed and stained. Stained cells were counted by light microscopy in nine randomly chosen high-power fields (x 200). Images were captured by a Photometrics Sensys CCD camera and imported into IP Labs image analysis software package. Columns, mean from three independent experiments; *bars*, ± SD.

To determine the effects of IR-induced VEGF on GBM tumor cell motility, we performed invasion assays on Matrigel-coated membranes and migration assays on uncoated membranes. Because VEGF expression after IR was higher in U251 than in LN18, we used the U251 glioma cell line for motility assays. IR-CM was used as a chemoattractant for the invasion and migration assays (Figure 3). The invasion rate of U251 glioma cells was increased with IR-CM (334.2 ± 46% of control; p = 0.002). IR-CM also enhanced U251 glioma cell migration rates (174.9 ± 11.4% of control; p = 0.001). Invasion and migration rates were also increased when we used the recombinant VEGF_165 _(5 ng/mL) as a positive control (296 ± 8.24% and 213.98 ± 49.7% of control, respectively). There were no statistically significant differences in invasion or migration rates between IR-CM and VEGF_165 _groups (p = 0.119). These data suggest that IR-induced VEGF in IR-CM is sufficient to cause the increased invasion and migration seen in the recombinant VEGF.

To assess the importance of the effect of VEGF in the tumor-conditioned medium, VEGF neutralizing antibody was added, and the motility assay was repeated. Various concentrations of anti-VEGF antibody (anti-human VEGF_165_; 1, 10 and 20 μg/mL) were added to IR-CM, and the invasion and migration assays were performed as described above. Compared to IR-CM, there was a significant reduction of U251 glioma cell motility when anti-VEGF antibody was added to IR-CM (Figure 3B). Invasion rates were significantly decreased by adding anti-VEGF antibody to IR-CM, and their diminutions were correlated with concentration of antibody (135.9 ± 4.8% of control with 10 μg/mL of anti-VEGF antibody, and 105.7 ± 14% of control with 20 μg/mL of anti-VEGF antibody). Enhanced migration rates observed with IR-CM were nullified with 10 μg/mL of antibody (109.9 ± 15.5% of control, p = 0.348), and increasing VEGF antibody up to 20 μg/mL did not further decrease the migration rates (118.2 ± 21.6% of control, p = 0.612). These data indicate that VEGF is necessary for causing the increased invasion and motility seen from tumor-conditioned medium and can be inhibited by a neutralizing antibody.

### Proliferation assay

**Figure 4:**
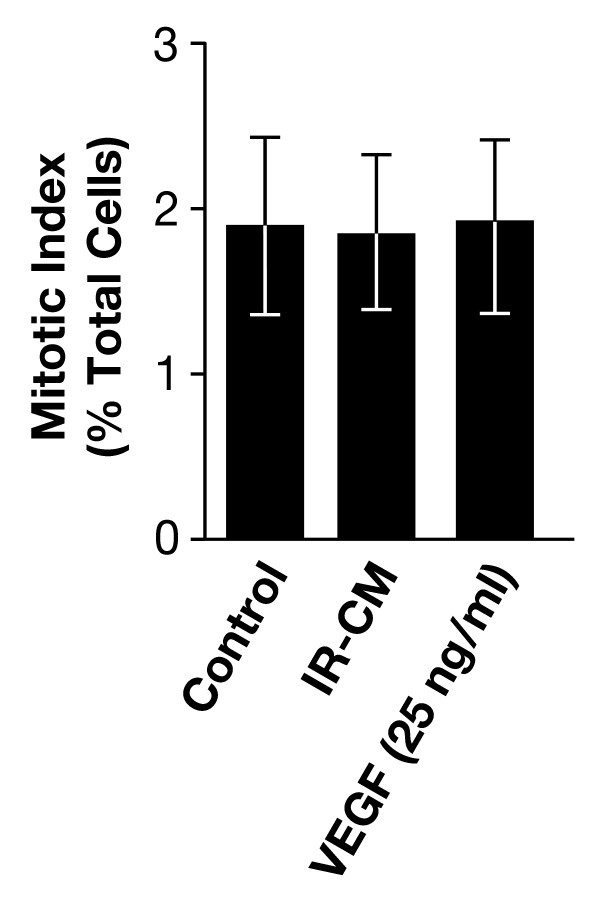
**Influence of irradiated conditioned medium (IR-CM) and VEGF on the proliferation of U251 glioma cell**. U251 GBM glioma cells were incubated for 22 h with conditioned medium or VEGF_165 _(25 ng/mL) contained medium. Evaluation of cells in mitotic phase was performed using flow cytometry. Samples were fixed, stained with propidium iodide, and analyzed using flow cytometry. Mitotic cells were distinguished from G_2 _cells, and the mitotic index was determined according to the expression of phosphorylated histone H3 as detected in the 4 N DNA content population by the flow cytometric method of *Xu et al*. [23]. The data represent the mean of three independent experiments; *bars*, ± SD.

To determine whether the enhanced GBM cellular motility was due to an increased number of cells secondary to the mitogenic effects of VEGF, we measured the mitotic index after the addition of IR-CM or VEGF stimulation of proliferation of GBM tumor cells using the method of Xu et al. [24]. This assay determines the percentage of mitotic cells in the 4 N population according to the flow cytometric analysis of phosphorylated histone H3, which is specifically expressed in mitotic cells. After 22 h of exposure to IR-CM or VEGF (25 ng/ml), there was no change in the mitotic index of U251 cells in the control, IR-CM and VEGF groups (Figure 4). These data indicate that VEGF in conditioned medium significantly enhances the migration and invasion activity of U251 glioma cells, and the effect is not due to an increase in proliferation.

### Immunoblotting

**Figure 5:**
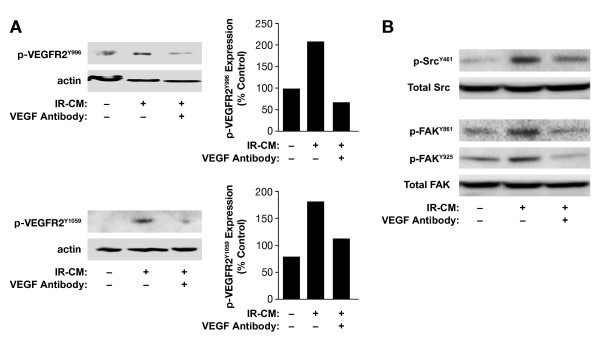
**The effects of irradiated conditioned medium (IR-CM) on activation of kinases in glioma cells**. After 16 h incubation of GBM glioma cells with IR-CM ± monoclonal anti-human VEGF_165 _antibody (R&D System), cells were harvested, processed to make lysates and the supernatant was collected. Equal quantities of protein were separated by SDS/PAGE and transferred to PVDF membrane. The membranes were blocked with 5% dried non-fat milk for 1 h at room temperature and incubated with the desired primary antibody diluted 1:1000 in blocking buffer overnight at 4 C. Membranes were probed with antibodies to phospho VEGFR2^Y996^, phospho VEGFR2^Y1056^, Src^Y461^, phospho FAK^Y861 ^and phospho FAK^Y925^. Primary antibody incubation was followed by incubation with a horseradish peroxidase-conjugated secondary antibody in blocking buffer for 2 h at room temperature. Proteins were visualized with electrochemiluminescence detection reagents and detected by autoradiography. (**A**) IR-CM effect on phosphorylation of VEGFR2 in U251 glioma cells. (**B**) IR-CM with/without VEGF antibody (10 μg/ml) effect on the phosphorylation of Src and FAK in U251 glioma cells. Blots are representative images of three replicates.

In tumor cells, motility is regulated partly by the activation of Src and FAK. A variety of factors are known to induce Src and FAK activation. VEGF is one of the molecules that can stimulate the phosphorylation of FAK through Src family activation [19,20]. To determine whether Src and/or FAK can be activated when glioma cells are treated with conditioned medium, first we investigated the activation status of VEGF receptor 2 (VEGFR2) after treatment with IR-CM. As shown in Figure 5A, treatment of U251 glioma cells with IR-CM enhanced phosphorylated VEGFR2 at both Y996 and Y1059 (210% of control and 187% of control, respectively). Increased phosphorylation of VEGFR2 was mitigated by adding VEGF antibody (10 μg/ml) in IR-CM.

To determine the effects of VEGF in IR-CM on downstream signaling of VEGFR2, we investigated the status of Src and FAK phosphorylation with IR-CM treatment. In Figure 5B, treatment of U251 glioma cells with IR-CM enhanced phosphorylation of Src kinase at Y461. Moreover, after 16 h of incubation of GBM glioma cells with IR-CM, U251 cells also expressed increased phosphorylation of FAK at both Y861 and Y925. To determine whether the enhancement of phosphorylation of Src and FAK in response to IR-CM was due to the effects of VEGF in IR-CM, anti-VEGF antibody was added to IR-CM. Anti-VEGF antibody in IR-CM effectively blocked Src and FAK phosphorylation.

Taken together, our data show VEGF in IR-CM can phosphorylate VEGFR2, leading to a VEGFR2-mediated downstream signaling (Src and FAK) cascade, thereby mediating enhanced cellular invasion and migration in GBM tumor cells.

## Discussion

Cytokines are released in response to a diverse range of cellular stresses such as infection, inflammation and injury, and regulate a variety of cellular functions. It has been reported that alteration of cytokines can change cellular interactions [8,9,12,13,25-28]. VEGF is an important angiogenic factor and induces a potent mitogenic signal for endothelial cells by binding VEGFRs on endothelial cells. Expression of VEGFRs, however, has also been identified in other cell types, including glioblastoma cell lines [29]. These data suggest that, in addition to angiogenic function, VEGF may affect the function of cancer cells that express VEGFRs [10,12,13].

In the present study, we evaluated the alteration of the extracellular VEGF (tumor-derived) concentration in two GBM cell lines (U251 and LN18) in response to a range of radiation doses (0.5-10 Gy). VEGF concentration in each cell line after radiation increased and showed a peak level at conventional daily radiation doses (1 ~ 3 Gy) (Figure 1). With higher doses, however, we found that VEGF concentration did not further increase. Our results are similar to another study, which showed increased VEGF levels in conditioned medium 24 h after radiation (0 *vs*. 5, 10 and 20 Gy) but the increase did not occur in a radiation dose-dependent manner [8]. Moreover, increased VEGF levels in IR-CM resulted from radiation-induced increased VEGF transcription in glioma cells (Figure 2). These results suggest that glioma cells produce and secrete VEGF after a conventional dose of radiation.

VEGF has been reported to regulate the functions of endothelial cells by binding VEGFRs on the cells. In addition to their expression on endothelial cells, VEGFRs have also been identified on hematopoietic origin cells and human cancer cells [9,12,13,16]. Damiano et al. [30] report the expression of VEGFRs on glioma cells and their signaling activity in conjunction with the epidermal growth factor receptor. Induction of cell motility in response to mitogenic factors such as VEGF is a tightly regulated process, requiring the coordination of a complex set of signals involving the extracellular matrix, integrins and the actin cytoskeletal-associated motile apparatus. Specifically, VEGFR2 activation results in the activation of Src and FAK.

In this study, we evaluated the effect of IR-CM or VEGF on the motility of glioma cells. As stated above, VEGF showed its enhanced migratory effects on glioma cells; both the invasion and migration index were increased in our study. Interestingly, we noticed that the glioma cell motility assay with IR-CM also resulted in an increase of the invasion and migration index; VEGF antibody, however, attenuated the migration activity in IR-CM (Figure 3). These data suggest that increased VEGF in IR-CM is necessary for increasing invasion and motility. These results are consistent with other data that have demonstrated the enhanced migratory effects of VEGF on various types of human cancer cells [9-13,16].

To investigate the mechanism responsible for mediating the VEGF in IR-CM-induced glioma cell motility, we surveyed VEGFR2-mediated downstream signaling pathways. VEGF has been reported to be capable of activating additional kinases, which play an important role in cell motility [11,18,19,21]. First, the Src are non-receptor tyrosine kinases, ubiquitously expressed in cells and involved in the cellular motility pathway. VEGF-induced Src activation and signaling also has been reported and is associated with poor prognosis in cancer patients [10,11,20,31]. Second, FAK is a widely expressed cytoplasmic protein tyrosine kinase that is phosphorylated in response to various stresses, and it plays an important role in controlling several fundamental cellular biological functions, including cell motility [32]. Glioma cells with low levels of phosphorylated FAK show motility arrest [33]. Interestingly, VEGF stimulates the tyrosine phosphorylation of FAK [19,20]. The phosphorylated FAK is associated with increased formation of stress fibers, recruitment of FAK to new focal adhesions and increased cell motility. It is also well known that FAK activation is closely related to Src activity [34]. Although the mechanism underlying VEGF-stimulated Src and FAK phosphorylation is still under the evaluation, data support the concept that cell motility is regulated by VEGF-mediated VEGFR2 activation and interaction with its downstream protein kinases, including Src and FAK [20].

Lesslie et al. [10] and Munshi et al. [11] reported VEGF-activated Src in human cancer cells. These results were consistent with data in this study. Glioma cells treated with VEGF showed enhanced Src activity. We also noticed that glioma cells treated with IR-CM showed increased Src and FAK expression. Enhanced kinase activities in glioma cells, however, were attenuated by adding VEGF antibody to IR-CM. These results suggest that radiation-induced VEGF in glioma cells might account for activation of Src and FAK, thereby enhancing cellular motility.

The data presented here indicate that glioma tumor cells produce VEGF after a conventional dose of radiation and, moreover, show that radiation-induced VEGF affects tumor cell motility by activating Src and FAK kinase. The studies described here addressed only the effect of radiation-induced VEGF on glioma cells *in vitro*. These findings support a model in which tumor-derived VEGF induced by radiation is both necessary and sufficient to increase tumor cell motility. Microscopically scattered glioma cells around a gross tumor area could be affected by radiation-induced VEGF during and after treatment. This may provide an additional mechanistic rationale to explain the frequent tumor recurrences seen with GBM after radiation therapy. It also suggests the potential to improve outcomes by combining radiation therapy with anti-VEGF agents.

## Abbreviations

Act D: actinomycin D; CM: conditioned medium; CT: computed tomography; DMEM: Dulbecco's modified eagle medium; ELISA: enzyme-linked immunosorbent assay; FAK: focal adhesion kinase; FBS: fetal bovine serum; GAPDH: glyceraldehyde-3-phosphate dehydrogenase; GBM: glioblastoma multiforme; IR: irradiation; IR-CM: irradiated-conditioned medium; MR: magnetic resonance; PBS: phosphate-buffered saline; PET: polyethylene teraphthalate; PVDF: polyvinyldene fluoride; RT: radiation therapy; SD: standard deviation; SDS/PAGE: sodium dodecyle sulfat/polyacrylamide gel electrophoresis; Src: Src kinase; VEGF: vascular endothelial growth factor; VEGFRs: vascular endothelial growth factor receptors

## Competing interests

The authors declare that they have no competing interests.

## Authors' contributions

WJK, PJT, and KC designed and planned the experiments. WJK performed the experiments. WJK, PJT and KC evaluated the data and wrote the manuscript. All co-authors approved the manuscript.
